# Interests and Preferences in Programs to Improve Health Among Men With or at Risk for Type 2 Diabetes in Racial and Ethnic Minority Groups, 2019

**DOI:** 10.5888/pcd22.240268

**Published:** 2025-01-09

**Authors:** LaShonda Hulbert, Yvonne Mensa-Wilmot, Stephanie Rutledge, Michelle Owens-Gary, Renée Skeete, Michael J. Cannon

**Affiliations:** 1 Division of Diabetes Translation, Centers for Disease Control and Prevention, Atlanta, Georgia; 2 Office of Policy, Performance, and Evaluation, Centers for Disease Control and Prevention, Atlanta, Georgia; 3 Sapodilla Group, LLC, Atlanta, Georgia

## Abstract

**Introduction:**

Men in racial and ethnic minority groups are less likely than non-Hispanic White men to participate in programs designed to improve health, despite having a higher prevalence of type 2 diabetes. We sought to understand 1) the interests and preferences of racial and ethnic minority men, with or at risk for type 2 diabetes, in programs designed to improve health and 2) factors that influence participation and health practices.

**Methods:**

We designed a 43-question web-based survey on facilitators and barriers to participation in a healthy living program. The survey was administered from August 27, 2019, through September 3, 2019. Our analytic sample consisted of 1,506 men at risk for or diagnosed with type 2 diabetes in racial and ethnic minority groups. We conducted descriptive and regression analyses of survey data.

**Results:**

Most men (59%) were interested in participating in a healthy living program and/or program elements such as incentives (67%), male-specific health topics (57%), and the inclusion of family (63%). Flexibility was important, since “exercising when it is convenient for me” was the most frequently selected facilitator of physical activity and “the hours were inconvenient” was identified as a challenge in previous programs. Men in this survey were significantly more likely to be interested in participating in a health improvement program for several reasons, including if they were physically active 150 minutes or more per week (vs not) (adjusted odds ratio [AOR] = 2.2; 95% CI, 1.6–3.0) and had previously been in a healthy living program (vs not) (AOR = 1.5; 95% CI, 1.1–2.1).

**Conclusion:**

Our findings can be useful for recruiting and retaining racial and ethnic minority men with or at risk for type 2 diabetes in programs designed to improve health and ultimately reduce disparities in the prevalence of diabetes.

**Summary**

**What is already known on this topic?**

Men in racial and ethnic minority groups are less likely than non-Hispanic White men to participate in diabetes prevention and management programs, despite having a higher prevalence of type 2 diabetes. Research is limited on men’s perceptions of lifestyle modification programs.

**What is added by this report?**

We identified characteristics and programmatic elements that might encourage men in racial and ethnic minority groups to participate in programs designed to improve their health.

**What are the implications for public health practice?**

Tailoring a program to the interests and preferences of men in racial and ethnic minority groups — with or at risk for type 2 diabetes — could lead to their increased participation in diabetes prevention and management programs.

## Introduction

Racial and ethnic disparities exist in the prevalence of diabetes in the US: The prevalence is higher among American Indian or Alaska Native men (13.4%), Asian men (10.6%), Black or African American men (11.5%), and Hispanic men (12.2%) than among non-Hispanic White men (7.7%) (1).

For people who have diabetes, diabetes self-management education and support services provide skills training, education, and support (2). For people at risk for type 2 diabetes, the National Diabetes Prevention Program (National DPP) lifestyle change program (LCP) is a year-long intervention designed to prevent or delay the onset of type 2 diabetes through moderate weight loss, healthy eating, physical activity, and stress management (3). However, men infrequently participate in diabetes education and prevention interventions (4,5). Likely contributors to men’s infrequent participation include financial challenges, a lack of access to quality care, lack of access to transportation, and a lack of social support, all of which have been reported as barriers to healthy living for men (6,7).

To encourage men’s participation in programs to improve health, studies have proposed enrolling all-male cohorts, developing programmatic content that is relevant and appealing to men, and recruiting male facilitators (8–10). Nevertheless, few studies have captured data from a diverse group of men regarding their interests and preferences in programs to improve their health (11,12). To help fill these gaps, we conducted a survey to capture interests and preferences in various elements of a healthy living program as well as health practices of racial and ethnic minority men at risk for or diagnosed with type 2 diabetes.

## Methods

### Survey development

The Centers for Disease Control and Prevention partnered with the National Association of Chronic Disease Directors to develop and distribute a 43-question survey on a priori knowledge of potential facilitators and barriers to participation in a program designed to improve health. The web-based survey was administered from August 27, 2019, through September 3, 2019. 

The survey protocol, informed consent, sampling design, and questionnaire were approved by Sterling Institutional Review Board (IRB ID-7292). We used a nonprobability quota sample drawn from an opt-in consumer panel provided by Dynata, a market research firm. The sampling frame included 3,000 men in the US from various regions and education and income levels and reached 1,506 men. Participants consented to the study when signing up and could exit the survey at any time. The final data set was postweighted to reflect the 2019 US adult male population per race and ethnicity (13).

### Survey sample

Participants were invited to take the survey if they self-identified as adult males (aged ≥18 y) living in the US, at risk for or diagnosed with type 2 diabetes, and members of the following racial or ethnic minority groups: American Indian or Alaska Native, Asian, Black or African American, Hispanic, Native Hawaiian or Pacific Islander, or multiple races. Participants were considered at risk for type 2 diabetes if they reported 1 or more of the following: diagnosed hypertension or prediabetes, a family history of type 2 diabetes, physical inactivity (<150 minutes of moderate-vigorous physical activity per week), aged 45 years or older, or a body mass index (BMI) of 23.0 or more for self-reported Asian race and a BMI of 25.0 or more for all other races and ethnicities. The unweighted sample consisted of 1,506 men. Participants received a monetary incentive for participation.

### Measures

Using the 2019 Behavioral Risk Factor Surveillance System (BRFSS) survey as a guide, we adapted validated measures, including time since the last doctor visit, health history, employment status, marital status, ethnicity, race, annual household income, and education level (14). We also used BRFSS measures to capture data on height and weight (to calculate BMI as weight in kilograms divided by height in meters squared), health history, and physical activity status. We developed survey items to capture data on location of primary residence and language spoken at home by consulting with subject matter experts and census data. We developed questions about interests and preferences for healthy living programs and piloted the full instrument with a small group (n = 5) from the respondent population.

We recoded age into 4 categories: 18 to 24, 25 to 44, 45 to 64, and 65 years or older. Annual household income was classified in 4 categories: less than $20,000, $20,000 to $49,999, $50,000 to $99,999, and $100,000 or more. We recoded the variable on last doctor visit in 5 categories: within the last 3 months, more than 3 months ago but less than 6 months ago, more than 6 months ago but less than 12 months ago, 12 months ago or longer, and “I don’t know.” To capture data on ethnicity, participants were asked, “Are you of Hispanic, Latino, or Spanish origin?” and answers were yes or no. Participants who said yes were included as Hispanic, regardless of race. To capture data on race, participants were asked “What is your race?” and answers included American Indian or Alaska Native, Asian, Black or African American, Native Hawaiian or Pacific Islander, or White. For those who selected more than 1 race we used the variable “multiple races.” We excluded from the survey participants who identified as non-Hispanic White.

**Interest in healthy living program elements. **Six questions assessed interest in elements of a healthy living program. These questions were prefaced by, “Would you be interested in participating in . . .” Responses consisted of a 5-answer Likert-type scale: “yes, definitely,” “yes, probably,” “I’m not sure,” “no, probably not,” and “no, definitely not.” We recoded these into 3 categories (yes, “I’m not sure,” and no).

**Facilitators to participation in healthy living programs.** Questions to determine whether certain elements would increase the likelihood of program participation were prefaced by, “Would you be more likely to participate . . . ” For men who indicated they spoke a language other than English at home (n  =  616), we included an additional question about using program materials in the language spoken at home. We recoded the 5-answer Likert-type scale (“yes, definitely,” “yes, probably,” “I’m not sure,” “no, probably not,” and “no, definitely not”) into 3 categories (yes, “I’m not sure,” and no).

**Healthy living program design preferences.** Participants who answered “yes, definitely” or “yes, probably” to the question “Would you be interested in participating in a group session on healthy living?” were asked 4 questions about program design preferences (n  =  897). These questions asked about preferred frequency of sessions, distance willing to travel, structure (structured vs informal), and setting (eg, classroom vs barber shop). The question, “What is the farthest you would be willing to travel to attend a group session on healthy living? (Assume the program is free and offered at a time when you are available)” was recoded from 6 answer choices to 5 by combining “between 20 to 60 miles” with “more than 60 miles.”

**Health practices.** Participants were asked, “Do you usually engage in physical activity for at least 150 minutes (2.5 hours) per week? Physical activity is any activity that speeds up your heart rate and breathing, such as walking at a brisk pace, running, cycling, playing basketball, swimming, etc.” Those who responded yes were asked to select what helped them maintain that level of physical activity. Those who answered no or “I don’t know” were asked to select what limited them from reaching the recommended physical activity level.

**Previous experience in formal programs to improve health.** The men who had previously participated in a health improvement program (n  =  460) were asked to identify challenges they encountered in those programs. They were asked, “Thinking of the formal programs you have previously participated in that have to do with improving your health, what problems or issues did you encounter with these programs?”

### Data analysis

We used cross-tabulations to produce a descriptive analysis of the participants, their interests and preferences in a healthy living program, and their health practices. We used χ^2^ tests to identify differences in interests and preferences among racial and ethnic groups; *P*  < .05 was considered significant.

We used multiple logistic regression to determine the association between the characteristics of the men and their health practices with the outcome: interest in participating in a healthy living program. We recoded the outcome variable, “Would you be interested in participating in a group session on healthy living?” into a dichotomous response (yes/no) to conduct the analysis. We included variables such as age, race and ethnicity, education level, and physical activity status to determine any interaction between them and the outcome. We used SPSS Statistics Subscription version 1.0.0.1406 (IBM Corp) to conduct the analysis in 2023.

## Results

Survey participants (N = 1,506) more frequently were aged 45 to 64 years (42.9%), were Hispanic (44.4%), had a BMI of 30.0 or more (48.8%), visited a doctor within the last 3 months (45.5%), were college graduates (48.0%), and reported an annual household income of $50,000 to $99,999 (34.5%) (Table 1). By design, approximately half of the sample had been diagnosed with type 2 diabetes (49.7%), and the rest were at risk for type 2 diabetes (50.3%). Most participants reported engaging in physical activity for at least 150 minutes per week (65.2%), receiving their health information from a doctor or doctor’s office (54.2%), and living in small cities, suburban areas, or large towns (53.8%). Combining responses from the men diagnosed with type 2 diabetes and those at risk for type 2 diabetes did not meaningfully change the results.

**Table 1:** Demographic Characteristics of Participants (N = 1,506) in a Survey of Men at Risk for or Diagnosed With Type 2 Diabetes in Racial and Ethnic Minority Groups^a^

Characteristic	No. (%)^b^
**Age, y**	
18–24	53 (3.5)
25–44	448 (30.3)
45–64	650 (42.9)
≥65	355 (23.4)
**Race and ethnicity**	
American Indian or Alaska Native	27 (1.7)
Asian	236 (15.2)
Hispanic	581 (44.4)
Native Hawaiian or Pacific Islander	16 (1.0)
Non-Hispanic Black or African American	577 (32.0)
Multiple races	70 (5.6)
**Diabetes status**	
Diagnosed with type 2 diabetes	750 (49.7)
At risk for type 2 diabetes	756 (50.3)
**Do you engage in physical activity for at least 150 minutes per week?^c^**	
Yes	981 (65.2)
No/don’t know	525 (34.8)
**Body mass index (BMI)** d	
Underweight (<18.5)	9 (0.6)
Normal weight (18.5–24.9)	178 (11.7)
Overweight (25.0–29.9)	585 (38.9)
Obese (≥30)	734 (48.8)
**About how long has it been since you last visited a doctor for a routine check-up?**	
Within the last 3 months	692 (45.5)
More than 3 months but less than 6 months ago	327 (22.0)
More than 6 months ago but less than 1 year ago	312 (20.8)
12 months ago or longer	144 (9.7)
I don’t know	31 (2.0)
**What is the highest grade or year of school you completed?**	
Some school^e^ or never attended school	58 (3.9)
High school graduate or GED	251 (16.4)
Some college or technical school	486 (31.8)
College, 4 years or more (graduate)	711 (48.0)
**Employment status**	
Employed for wages	860 (57.3)
Unemployed	99 (6.6)
Other (including student, homemaker)	38 (2.5)
Retired	414 (27.5)
Unable to work	95 (6.1)
**Marital status**	
Married	827 (55.5)
Never married	401 (26.2)
Member of an unmarried couple	75 (5.0)
Other (widowed, divorced, separated)	203 (13.3)
**Which of the following best describes the location of your primary residence?**	
Large city	557 (36.7)
Small city, suburban area, or large town	809 (53.8)
Village or rural	134 (9.1)
A reservation	6 (0.4)
**Annual household income, $**	
<20,000	201 (13.1)
20,000–49,999	361 (23.6)
50,000–99,999	511 (34.5)
≥100,000	433 (28.9)
**Do you speak a language other than English at home?**	
Yes	616 (43.8)
No	890 (56.2)
**Have you ever participated in any formal programs aimed at improving your health?** f	
Yes	462 (30.6)
No	1,044 (69.4)
**Where do you get information about health-related activities? (Select all that apply)** g	
Doctor or doctor’s office	817 (54.2)
The internet or social media	607 (40.2)
Television	482 (31.3)
A friend, family member, or relative	450 (29.8)
Gym or health club	290 (19.0)
Pharmacy or pharmacist	198 (13.1)
Somewhere else	128 (8.6)
Radio or podcasts	124 (8.2)
A community organization	113 (7.5)
Local government	77 (5.1)
Church	76 (4.9)
School	63 (4.2)
Medicine men	60 (4.0)
Barbershop or hair salon	42 (2.7)
Sweat lodges	22 (1.6)
None of the above	115 (7.7)

Abbreviation: GED, General Educational Development.

a Data source: The Centers for Disease Control and Prevention partnered with the National Association of Chronic Disease Directors to develop and distribute a 43-question survey from August 27, 2019, through September 3, 2019. Percentages may not add to 100 because of rounding.

b Weighted percentage.

c Physical activity is any activity that speeds up your heart rate and breathing, such as walking at a brisk pace, running, cycling, playing basketball, swimming, etc.

d Calculated by using self-reported height and weight data.

e Some school includes kindergarten through grade 11.

f A formal program could include a group education course, one-on-one sessions with a health coach, a team weight-loss competition, or another similar program.

g Adds to >100% because >1 answer could be selected; answers are not mutually exclusive.

### Interest in healthy living program elements

Many men indicated interest in participating in a group session on healthy living (59.3%) (Table 2), despite 69.4% having never participated in a formal health improvement program (Table 1). A slightly smaller percentage of men were interested if the sessions were held online (55.7%). The men showed more interest in program elements such as incentives for losing or maintaining weight (67.3%) and programs that include families (63.3%) (Table 2). Working with a personal health coach (58.5%) and male-centered topics such as erectile dysfunction and diabetes (57.1%) were also of interest. Among men (n = 1,340) who had a BMI of 25.0 or more (or ≥23.0 for Asian men), approximately half (51.4%) showed interest in a program in which they could compete on a team to lose weight.

**Table 2:** Interest in Program Elements and Facilitators to Participation in a Program Designed to Improve Health for Men at Risk for or Diagnosed With Type 2 Diabetes in Racial and Ethnic Minority Groups^a^^,^ b

Survey item	Total (N = 1,506)	AI/AN (n = 27)	Asian (n = 236)	Black (n = 577)	Hispanic (n = 581)	NH/PI (n = 16)	Multiple races (n = 69)	*P* value^c^
**Would you be interested in participating in a group session on healthy living?**								
Yes, definitely	426 (28.0)	2 (7.4)	47 (20.0)	193 (33.4)	170 (29.3)	3 (18.7)	11 (16.5)	<.001
Yes, probably	471 (31.3)	10 (37.0)	80 (33.9)	176 (30.5)	173 (29.8)	7 (43.7)	25 (36.5)	
I’m not sure	328 (22.0)	8 (29.6)	58 (24.3)	110 (19.1)	137 (23.6)	1 (6.3)	14 (20.0)	
No, probably not	172 (11.3)	4 (14.8)	35 (14.8)	69 (12.0)	53 (9.1)	2 (12.5)	9 (12.9)	
No, definitely not	109 (7.5)	3 (11.1)	16 (7.0)	29 (5.0)	48 (8.2)	3 (18.8)	10 (14.1)	
**Would you be interested in participating in a group session on healthy living that is held online?**								
Yes	844 (55.7)	15 (55.6)	113 (48.0)	350 (60.6)	322 (55.4)	10 (62.5)	34 (49.4)	.10
I’m not sure	322 (21.4)	4 (14.8)	57 (24.0)	117 (20.3)	128 (22.0)	2 (12.5)	14 (20.0)	
No	340 (22.9)	8 (29.6)	66 (27.9)	110 (19.1)	131 (22.6)	4 (25.0)	21 (30.6)	
**Would you be interested in participating in a group session on healthy living that provides information about ways to prevent or delay erectile dysfunction? (Assume the sessions are free and held at a time when you are available.)^d^**								
Yes	866 (57.1)	12 (44.4)	117 (49.8)	364 (63.1)	332 (57.1)	8 (50.0)	33 (48.8)	.003
I’m not sure	301 (20.2)	9 (33.3)	60 (25.3)	93 (16.2)	125 (21.5)	2 (12.5)	12 (16.7)	
No	339 (22.6)	6 (22.2)	59 (24.9)	120 (20.7)	124 (21.4)	6 (37.5)	24 (34.5)	
**Would you be interested in participating in a program that offers incentives (nonfinancial or financial) for losing and/or maintaining your weight?**								
Yes	1,018 (67.3)	19 (69.2)	149 (63.0)	408 (70.7)	389 (67.0)	13 (81.3)	40 (58.8)	.22
I’m not sure	251 (16.7)	3 (11.5)	38 (16.1)	95 (16.4)	99 (17.0)	1 (6.3)	15 (21.2)	
No	237 (16.0)	5 (19.2)	49 (20.9)	74 (12.9)	93 (16.0)	2 (12.5)	14 (20.0)	
**Would you be interested in participating in a program where you compete in a team to lose weight? (Assume this program is free to participate and held at a time when you are available.)^e^**								
Yes	691 (51.4)	12 (44.4)	103 (47.6)	273 (54.3)	270 (52.7)	9 (64.3)	24 (36.6)	.11
I’m not sure	269 (20.1)	4 (14.8)	49 (22.9)	97 (19.3)	103 (20.0)	1 (7.1)	15 (22.0)	
No	380 (28.5)	11 (40.7)	64 (29.5)	133 (26.4)	140 (27.3)	4 (28.6)	28 (41.5)	
**Would you be interested in participating in a healthy eating program with your family, children, and/or those who live with you?**								
Yes	960 (63.3)	14 (51.9)	139 (59.0)	397 (68.9)	361 (62.1)	10 (62.5)	39 (56.5)	.008
I’m not sure	273 (18.5)	7 (25.9)	54 (22.7)	78 (13.5)	120 (20.7)	0	14 (20.0)	
No	273 (18.2)	6 (22.2)	43 (18.3)	102 (17.6)	100 (17.2)	6 (37.5)	16 (23.5)	
**Would you be interested in working with a personal health coach, that is, someone who can help you identify ways to incorporate healthy living in your life? (Assume the health coach is free.)**								
Yes	884 (58.5)	15 (55.6)	132 (55.9)	355 (61.5)	336 (57.9)	9 (56.3)	37 (54.1)	.74
I’m not sure	294 (19.7)	4 (14.8)	47 (20.1)	105 (18.2)	123 (21.1)	3 (18.8)	12 (17.6)	
No	328 (21.8)	8 (29.6)	57 (24.0)	117 (20.3)	122 (21.0)	4 (25.0)	20 (28.2)	
**Facilitators to participation**								
**Would you be more likely to participate in a group session on healthy living if the group was led by a man?**								
Yes	654 (43.1)	9 (33.3)	96 (40.6)	268 (46.5)	257 (44.2)	6 (37.5)	18 (25.9)	.02
I’m not sure	497 (33.2)	12 (44.4)	92 (38.9)	175 (30.3)	186 (32.0)	4 (25.0)	28 (41.2)	
No	355 (23.7)	6 (20.5)	48 (20.5)	134 (23.2)	138 (23.8)	6 (37.5)	23 (32.9)	
**Would you be more likely to participate in a group session on healthy living if the group was led by someone from your racial/ethnic group?**								
Yes	659 (43.2)	8 (29.6)	96 (40.6)	286 (49.6)	245 (42.2)	4 (25.0)	20 (29.4)	.002
I’m not sure	481 (32.0)	10 (37.0)	84 (35.8)	175 (30.3)	185 (31.8)	6 (37.5)	21 (30.6)	
No	366 (24.8)	9 (33.3)	56 (23.6)	116 (20.1)	151 (26.0)	6 (37.5)	28 (40.0)	
**Would you be more likely to participate in a program to improve your health if the program materials, such as flyers or videos, used examples and images of people from your racial/ethnic group?**								
Yes	725 (47.3)	9 (33.3)	100 (42.4)	328 (56.8)	263 (45.3)	5 (31.3)	20 (29.4)	<.001
I’m not sure	409 (27.2)	8 (29.6)	82 (34.9)	144 (24.9)	147 (25.3)	4 (25.0)	24 (34.1)	
No	372 (25.5)	10 (37.0)	54 (22.7)	105 (18.3)	171 (29.4)	7 (43.8)	25 (36.5)	
**Would you be more likely to participate in a program to improve your health if the program materials, such as flyers or videos, were provided in the language you speak at home?^f^**								
Yes	398 (64.2)	1 (50.0)	65 (55.8)	70 (78.4)	251 (64.5)	1 (25.0)	10 (63.2)	.02
I’m not sure	110 (18.0)	1 (50.0)	28 (23.9)	9 (10.8)	66 (17.0)	3 (75.0)	3 (21.1)	
No	108 (17.7)	0	24 (20.4)	9 (10.8)	72 (18.5)	0	3 (15.8)	

Abbreviations: AI/AN, American Indian or Alaska Native; NH/PI, Native Hawaiian or Pacific Islander.

a Data source: The Centers for Disease Control and Prevention partnered with the National Association of Chronic Disease Directors to develop and distribute a 43-question survey from August 27, 2019, through September 3, 2019. Percentages may not add to 100 because of rounding.

b Weighted percentage.

c χ^2^ tests were used for each variable to examine differences across categories; *P* < .05 is considered significant.

d The question asked was the following: “Men with diabetes are three times more likely to have erectile dysfunction (ED). Knowing this, would you be interested in participating in a group session on healthy living that provides information about ways to prevent or delay ED? (Assume the sessions are free and held at a time when you are available.)”

e Includes only participants with a BMI ≥23 for Asians and BMI ≥25 for all other races, ie, participants who are overweight. N’s for this question were the following: total (N  =  1,340); AI/AN (n  =  27); Asian (n  =  216); Black (n  =  503); Hispanic (n  =  513); NH/PI (n  =  14); multiple races (n  =  67).

f Includes only participants who selected yes to the question, “Do you speak a language other than English at home?” N’s for this question were the following: total (n  =  616); AI/AN (n  =  2); Asian (n  =  117); Black (n  =  88); Hispanic (n  =  389); NH/PI (n  = 4); multiple races (n  =  16).

### Facilitators to participation in healthy living programs

Approximately 43% said they would be more likely to participate in a healthy living program if the group was led by a man or by someone from their racial and ethnic group (Table 2). Nearly half (47.3%) said they would be more likely to participate if the program used materials with examples and images of people from their racial and ethnic group. For participants who indicated that they spoke a language other than English at home (n  =  616), 64.2% agreed that if the program materials were in the language they spoke at home, they would be more likely to participate.

### Healthy living program design preferences

A program that held sessions up to once a week was preferred (58.6%), compared with sessions held up to twice monthly (26.3%) or up to once monthly (9.9%) (Table 3). Almost one-quarter of men (23.3%) were willing to travel no more than 3 miles for a program, 36.1% were willing to travel no more than 5 miles, and 28.2% were willing to travel 5 to 20 miles. Men had a slight preference for informal and discussion-based sessions (41.7%) over those structured as a class (36.6%). Sessions held in an existing gathering space (eg, a community center, barbershop, coffee shop) were slightly preferrable (39.1%) to a classroom setup (27.6%), but one-third of the men had no preference for session location (33.3%).

**Table 3:** Preferences for the Design of a Healthy Living Program for Men at Risk for and Diagnosed With Type 2 Diabetes in Racial and Ethnic Minority Groups^a^^,^ b ^,^ c

Survey item	Total (N = 897)	AI/AN (n = 12)	Asian (n = 127)	Black (n = 369)	Hispanic (n = 343)	NH/PI (n = 10)	Multiple races (n = 36)	*P* value^d^
**Convenience (frequency and distance)**								
How often would you be willing to participate in a group session on healthy living?								
Up to once a week	525 (58.6)	9 (75.0)	72 (56.5)	215 (58.3)	201 (58.8)	8 (80.0)	20 (55.6)	.94
Up to twice a month	238 (26.3)	2 (16.7)	37 (29.0)	102 (27.5)	88 (25.7)	0	9 (24.4)	
Up to once a month	87 (9.9)	1 (8.3)	11 (8.9)	33 (9.1)	36 (10.4)	2 (20.0)	4 (11.1)	
Less than once a month	7 (0.8)	0	2 (1.6)	2 (0.6)	3 (0.8)	0	0	
No preference	40 (4.5)	0	5 (4.0)	17 (4.5)	15 (4.3)	0	3 (8.9)	
What is the farthest you would be willing to travel to attend a group session on healthy living? (Assume the program is free and held at a time when you are available.)								
NA (not willing to travel at all)	65 (7.0)	0	6 (4.9)	34 (9.1)	22 (6.3)	0	3 (8.7)	.003
No more than 3 miles	203 (23.3)	0	30 (23.6)	68 (18.5)	90 (26.3)	4 (40.0)	11 (30.4)	
No more than 5 miles	328 (36.1)	9 (75.0)	58 (45.5)	140 (38.0)	110 (32.2)	1 (10.0)	10 (28.3)	
Between 5 and 20 miles	250 (28.2)	3 (25.0)	30 (23.6)	96 (26.0)	105 (30.6)	5 (50.0)	11 (30.4)	
20 miles or greater	51 (5.4)	0	3 (2.4)	31 (8.4)	16 (4.6)	0	1 (2.2)	
**Structure (format and location)**								
Would you prefer to participate in a group session on healthy living that is structured and set up like a class, or informal and discussion based?								
Structured and set up like a class	327 (36.6)	3 (25.0)	46 (36.6)	133 (36.0)	131 (38.2)	2 (20.0)	12 (33.3)	.98
Informal and discussion based	374 (41.7)	6 (50.0)	55 (43.1)	152 (41.2)	141 (41.0)	5 (50.0)	15 (42.2)	
No preference	196 (21.7)	3 (25.0)	26 (20.3)	84 (22.7)	71 (20.8)	3 (30.0)	9 (24.4)	
Held in a classroom or another gathering space such as a community center, barbershop, or coffee shop? (Assume you are able to easily access any of these options.)								
Held in a classroom	247 (27.6)	4 (33.3)	34 (26.8)	101 (27.3)	97 (28.4)	2 (20.0)	9 (25.0)	.81
Held in an existing gathering space (a community center, a barbershop, a coffee shop, etc.)	351 (39.1)	5 (41.7)	59 (46.3)	138 (37.3)	138 (38.7)	4 (40.0)	12 (34.1)	
No preference	299 (33.3)	3 (25.0)	34 (26.8)	130 (35.4)	130 (32.9)	4 (40.0)	15 (40.9)	

Abbreviations: AI/AN, American Indian or Alaska Native; NA, not applicable; NH/PI, Native Hawaiian or Pacific Islander.

a Data source: The Centers for Disease Control and Prevention partnered with the National Association of Chronic Disease Directors to develop and distribute a 43-question survey from August 27, 2019, through September 3, 2019. Percentages may not add to 100 because of rounding.

b Includes only participants who selected “yes, definitely” or “yes, probably” to the question, “Would you be interested in participating in a group session on healthy living?”

c Weighted percentage.

d χ^2^ tests were used for each variable to examine differences across categories; *P* < .05 considered significant.

Some of the variables were significantly different across racial and ethnic groups (Table 2 and Table 3). However, the absolute differences in percentages were usually minor (<10%).

### Challenges in previous healthy living programs

**Figure 1:**
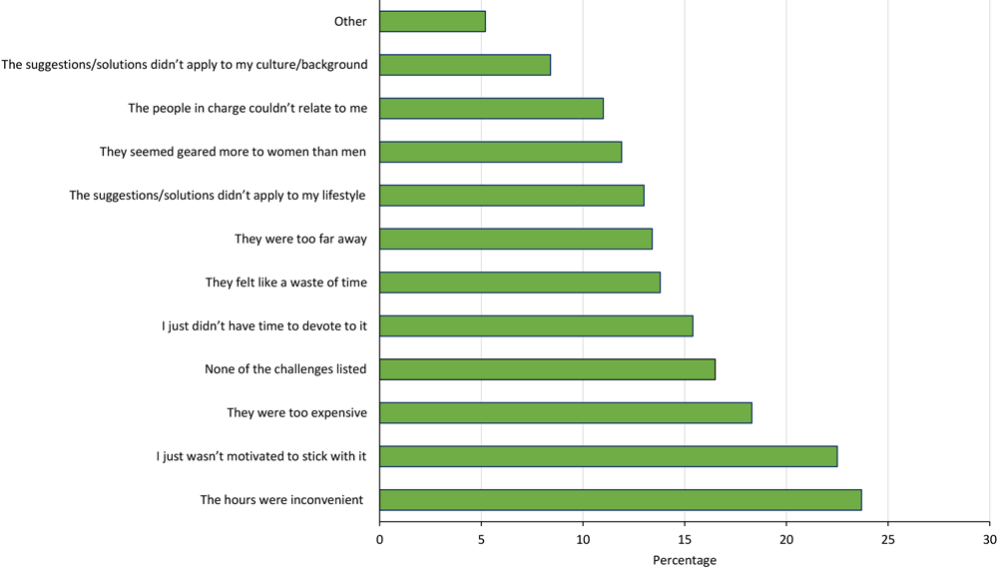
Challenges encountered in previous healthy living programs reported in a survey of men in racial and ethnic minority groups at risk for or diagnosed with type 2 diabetes (n = 462). “Other” challenges included distance (too far), program ended, problem with staff, cost and insurance issues, illness, and life circumstances. Participants could select multiple answers; percentages were weighted. Data source: 43-question survey developed and distributed (August 27, 2019–September 3, 2019) by the Centers for Disease Control and Prevention and the National Association of Chronic Disease Directors.

Some men (n  =  462) faced challenges in previous healthy living programs (Figure 1). The 3 most frequently noted challenges were inconvenient hours (23.7%), lack of motivation (22.5%), and program expense (18.3%).

**Table:** 

Reason	Percentage
The hours were inconvenient	23.7
I just wasn’t motivated to stick with it	22.5
They were too expensive	18.3
None of the challenges listed	16.5
I just didn’t have time to devote to it	15.4
They felt like a waste of time	13.8
They were too far away	13.4
The suggestions/solutions didn’t apply to my lifestyle	13.0
They seemed geared more to women than men	11.9
The people in charge couldn’t relate to me	11.0
The suggestions/solutions didn’t apply to my culture/background	8.4
Other	5.2

### Facilitators and barriers to a physical activity routine

**Figure 2:**
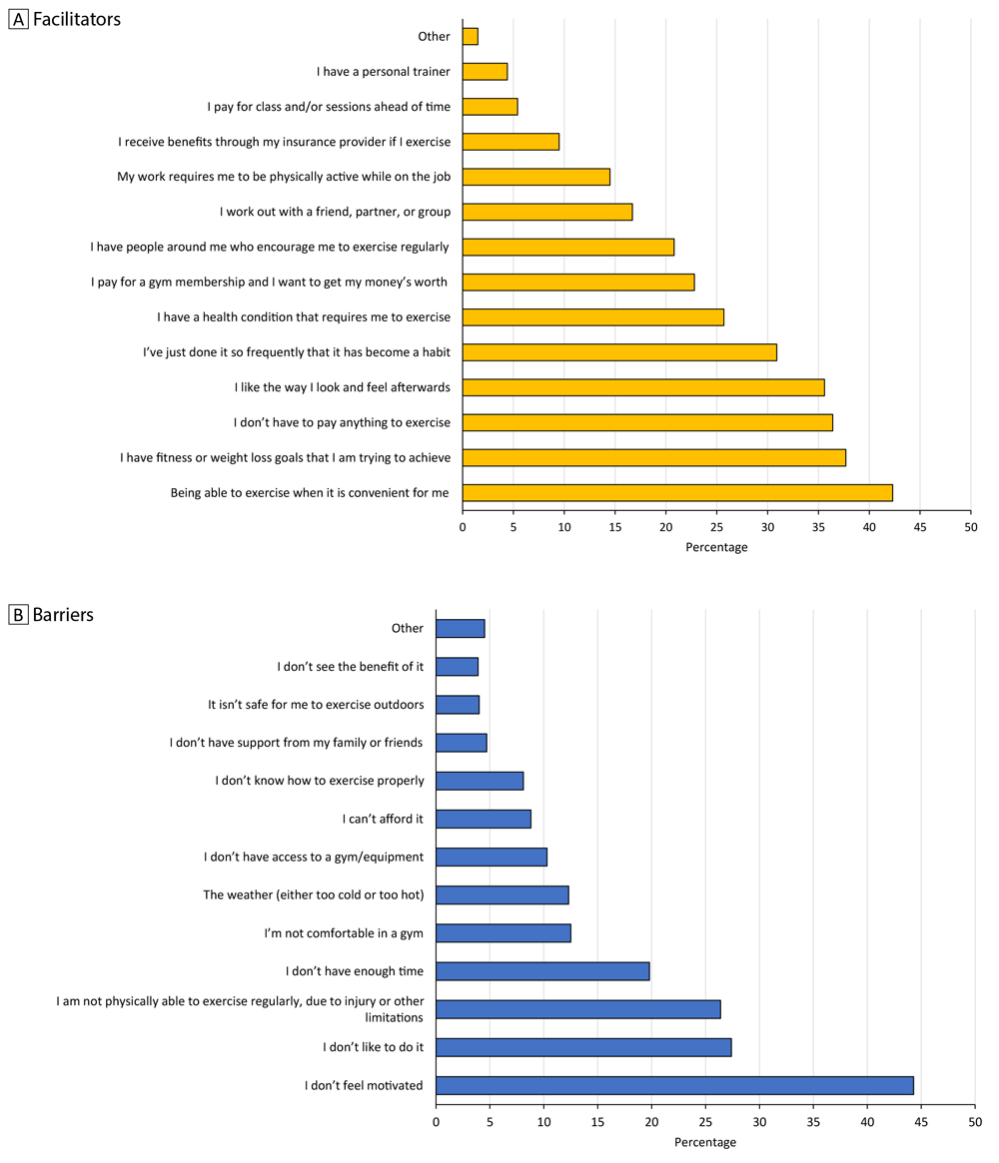
Facilitators and barriers to maintaining a physical activity routine reported in survey of men at risk for or diagnosed with type 2 diabetes in racial and ethnic minority groups. A. Facilitators to maintaining a physical activity routine among men (n = 981) who indicated that they engaged in physical activity for at least 150 minutes per week. “Other” facilitators included personal exercise equipment, pets, habit/lifestyle, and requirement of physical therapy. B. Barriers to maintaining a physical activity routine among men (n = 525) who indicated they do not or do not know if they engage in physical activity for at least 150 minutes per week. “Other” barriers were health, work conditions and/or schedule, lack of motivation, no babysitter, no reason given. Participants could select multiple answers; percentages were weighted. Data source: 43-question survey developed and distributed (August 27, 2019–September 3, 2019) by the Centers for Disease Control and Prevention and the National Association of Chronic Disease Directors.

Among men who reported engaging in physical activity for at least 150 minutes weekly (n  =  981), the most frequently selected facilitators for maintaining a physical activity routine were “Being able to exercise when it is convenient for me” (42.3%), “I have fitness or weight loss goals that I am trying to achieve” (37.7%), and “I don’t have to pay anything to exercise” (36.4%) (Figure 2A). The most frequently selected (n =  525) barriers to physical activity were “I don’t feel motivated” (44.3%), “I don’t like to do it” (27.4%), and “I am not physically able to exercise regularly, due to an injury or other limitations” (26.4%) (Figure 2B).

**Table:** 

Item	Percentage
**Facilitators**	
Being able to exercise when it is convenient for me	42.3
I have fitness or weight loss goals that I am trying to achieve	37.7
I don’t have to pay anything to exercise	36.4
I like the way I look and feel afterwards	35.6
I’ve just done it so frequently that it has become a habit	30.9
I have a health condition that requires me to exercise	25.7
I pay for a gym membership and I want to get my money’s worth	22.8
I have people around me who encourage me to exercise regularly	20.8
I work out with a friend, partner, or group	16.7
My work requires me to be physically active while on the job	14.5
I receive benefits through my insurance provider if I exercise	9.5
I pay for classes/sessions ahead of time	5.4
I have a personal trainer	4.4
Other	1.5
**Barriers**	
I don’t feel motivated	44.3
I don’t like to do it	27.4
I am not physically able to exercise regularly, due to injury or other limitations	26.4
I don’t have enough time	19.8
I’m not comfortable in a gym	12.5
The weather (either too cold or too hot)	12.3
I don’t have access to a gym/equipment	10.3
I can’t afford it	8.8
I don’t know how to exercise properly	8.1
I don’t have support from my family or friends	4.7
It isn’t safe for me to exercise outdoors	4.0
I don’t see the benefit of it	3.9
Other	4.5

### Factors associated with interest in healthy living programs

The adjusted multivariate model (Table 4) showed that participants were significantly more likely to be interested in participating in a group session on healthy living if they 1) were aged 25 to 44 years (adjusted odds ratio [AOR] = 2.0; 95% CI, 1.2–3.3) or 45 to 64 years (AOR = 1.5; 95% CI, 1.0–2.3) compared with participants aged 65 years or older, and 2) had an annual household income of $50,000 to $99,999 (AOR = 1.5; 95% CI, 1.0–2.1) compared with participants with an annual household income of $100,000 or more. Men who were physically active at least 150 minutes weekly (AOR = 2.2; 95% CI, 1.6–3.0) and had previously participated in a program designed to improve their health (AOR = 1.5; 95% CI, 1.1–2.1) were also significantly more likely to be interested. Compared with the men who were employed, those who were unemployed (AOR = 0.4; 95% CI, 0.2–0.8), retired (AOR = 0.5; 95% CI, 0.3–0.7), and unable to work (AOR = 0.4; 95% CI, 0.2–0.8) were significantly less likely to be interested in program participation. Those men who had their last doctor visit more than 3 months but less than 6 months ago (AOR = 0.7; 95% CI, 0.5–1.0) and more than 12 months ago (AOR = 0.5; 95% CI, 0.3–0.8) were also significantly less likely to be interested compared with men who had seen a doctor within the last 3 months.

**Table 4:** Predictors of Interest in Participating in a Healthy Living Program for Men at Risk for or Diagnosed With Type 2 Diabetes in Racial and Ethnic Minority Groups^a^

Variable	No. (%)^b^	Unadjusted OR (95% CI) [*P* value^c^]	Adjusted OR (95% CI) [*P* value^c^]
**Age, y**			
≥65	249 (69.6)	1 [Reference]	1 [Reference]
45–64	538 (82.7)	1.1 (0.9–1.5) [.21]	1.5 (1.0–2.3) [.04]
25–44	391 (87.5)	1.8 (1.3–2.5) [<.001]	2.0 (1.2–3.3) [.01]
18–24	47 (88.5)	1.8 (0.7–4.2) [.18]	1.8 (0.6–5.5) [.28]
**Race and ethnicity**			
American Indian or Alaska Native	20 (73.1)	0.6 (0.2–1.5) [.35]	0.7 (0.3–1.9) [.54]
Asian	185 (78.6)	0.8 (0.5–1.1) [.23]	0.8 (0.5–1.3) [.41]
Black	479 (83.0)	1.1 (0.8–1.5) [.23]	1.1 (0.8–1.5) [.61]
Hispanic	480 (82.6)	1 [Reference]	1 [Reference]
Native Hawaiian or Pacific Islander	11 (68.8)	0.5 (0.1–1.4) [.21]	0.5 (0.1–1.5) [.20]
Multiple races	50 (72.9)	0.5 (0.3–0.9) [.04]	0.7 (0.4–1.3) [.26]
**Education**			
College, 4 years or more (graduate)	578 (81.2)	1 [Reference]	1 [Reference]
Some college or technical school	391 (80.8)	0.9 (0.7–1.2) [.71]	0.9 (0.6–1.2) [.56]
High school graduate or GED	209 (82.6)	1.1 (0.7–1.6) [.52]	1.1 (0.7–1.8) [.61]
Some school^d^ or never attended school	47 (81.0)	0.9 (0.5–1.9) [.94]	0.7 (0.3–1.6) [.48]
**Employment**			
Employed for wages	751 (87.3)	1 [Reference]	1 [Reference]
Unemployed	75 (76.8)	0.7 (0.4–1.2) [.24]	0.4 (0.2–0.8) [.008]
Other (including student, homemaker)	34 (89.5)	1.8 (0.6–5.1) [.23]	1.2 (0.4–3.8) [.73]
Retired	292 (70.3)	0.4 (0.3–0.5) [**<**.001]	0.5 (0.3–0.7) [<.001]
Unable to work	73 (76.1)	0.7 (0.4–1.1) [.16]	0.4 (0.2–0.8) [.006]
**Annual household income, $**			
≥100,000	347 (79.8)	1 [Reference]	1 [Reference]
50,000–99,999	424 (83.0)	1.1 (0.9–1.5) [.21]	1.5 (1.0–2.1) [.04]
20,000–49,999	291 (80.8)	0.9 (0.7–1.3) [.81]	1.2 (0.8–1.9) [.31]
<20,000	163 (80.7)	0.9 (0.6–1.3) [.80]	1.5 (0.9–2.7) [.15]
**Primary residence**			
Large city	465 (83.5)	1 [Reference]	1 [Reference]
Small city, suburban area, or large town	656 (81.0)	0.9 (0.7–1.2) [.84]	1.0 (0.7–1.3) [.83]
Village or rural	99 (73.0)	0.6 (0.4–0.8) [.01]	0.7 (0.4–1.0) [.10]
A reservation	5 (83.3)	1.1 (0.1–9.8) [.93]	1.0 (0.8–12.5) [.99]
**Marital status**			
Married	666 (80.5)	1 [Reference]	1 [Reference]
Never married	333 (83.0)	1.1 (0.8–1.6) [.28]	0.9 (0.6–1.3) [.47]
Member of an unmarried couple	62 (82.7)	1.0 (0.5–1.9) [.80]	1.1 (0.6–2.2) [.76]
Other (widowed, divorced, separated)	164 (80.8)	1.0 (0.6–1.4) [.95]	1.1 (0.7–1.7) [.73]
**Last doctor visit**			
Within the last 3 months	572 (82.7)	1 [Reference]	1 [Reference]
More than 3 months but less than 6 months ago	262 (80.1)	0.8 (0.6–1.1) [.40]	0.7 (0.5–1.0) [.04]
More than 6 months ago but less than 1 year ago	259 (83.0)	1.2 (0.8–1.6) [.28]	0.8 (0.5–1.2) [.33]
12 months ago or longer^e^	107 (74.3)	0.6 (0.4–0.9) [.03]	0.5 (0.3–0.8) [.002]
I don’t know	25 (80.6)	1.0 (0.4–2.5) [.96]	1.0 (0.3–2.7) [.96]
**Physically active ≥150 min per week**			
No/I don’t know	378 (71.9)	1 [Reference]	1 [Reference]
Yes	847 (86.3)	2.4 (1.8–3.1) [<.001]	2.2 (1.6–3.0) [<.001]
**Body mass index**			
Normal weight (18.5–24.9)	152 (85.2)	1 [Reference]	1 [Reference]
Underweight (<18.5)	8 (88.9)	2.1 (0.2–20.3) [.52]	1.0 (0.8–11.3) [.98]
Overweight (25.0–29.9)	471 (80.7)	0.9 (0.7–1.2) [.64]	0.8 (0.5–1.4) [.49]
Obese (≥30)	594 (80.7)	0.9 (0.7–1.1) [.56]	0.8 (0.5–1.3) [.35]
**Previous participation in a health program**			
No	822 (78.7)	1 [Reference]	1 [Reference]
Yes	403 (87.2)	1.8 (1.3–2.4) [<.001]	1.5 (1.1–2.1) [.01]

Abbreviation: GED, General Educational Development.

a Data source: The Centers for Disease Control and Prevention partnered with the National Association of Chronic Disease Directors to develop and distribute a 43-question survey from August 27, 2019, through September 3, 2019.

b Weighted percentage.

c *P* value calculated by using SPSS software multivariate logistic regression; *P* < .05 considered significant.

d Some school includes kindergarten through grade 11.

e Includes up to 5 years ago.

## Discussion

To our knowledge, this is the first large survey of men — with or at risk for type 2 diabetes in racial and ethnic minority groups — reporting their interests, preferences, and previous challenges in a program designed to improve their health.

Many men surveyed were interested in participating in a group session on healthy living, although most had never participated in one. This finding stands in contrast to the gap in the uptake of programs such as the National DPP LCP (15) and diabetes self-management education and support services (16). The men surveyed also expressed interest in participating in healthy living programs online, which highlights the importance of flexibility in program delivery and an opportunity to overcome transportation challenges. This finding is timely since, after the survey was administered, the COVID-19 pandemic occurred and most health promotion activities relied on virtual delivery (17,18). Several program elements were appealing to the men surveyed, such as the inclusion of family and incentives. This finding provides some evidence for including or bolstering these program elements — especially since chronic disease prevention and management programs that have participant incentives demonstrate greater reductions in bodyweight and BMI compared with programs that omit incentives (19). Additionally, some men were interested in a program that includes information on how to prevent or delay erectile dysfunction. To our knowledge this finding has not been reported in the literature.

We found that healthy living program sessions led by a man or someone from participants’ racial or ethnic group, and program materials that featured examples of people from their racial or ethnic group, were likely facilitators to program participation. This finding supports research showing that using male-centered topics (20,21) and the inclusion of culture (21–23) can motivate men in chronic disease prevention and management programs. Also, providing program materials in the language participants speak at home was preferred — which is promising since both diabetes prevention and management programs are offered in English and Spanish (2,15).

A program with more frequent (ie, weekly) versus less frequent sessions appealed to the men; frequent sessions are associated with success for participants in both the National DPP LCP (24) and diabetes self-management education and support services (25). Respondents preferred a short travel distance to the program site over a program that was farther away, a key factor in increasing the likelihood of engaging in physical activity (26). Finally, because survey participants slightly favored an informal and discussion-based session over a structured class, consulting men about format and location preferences may be beneficial since social and environmental challenges may inhibit motivation or ability to participate. These program design preferences point to a desire to participate in a program that men perceive as having a convenient and comfortable space, which is especially important since people in racial and ethnic minority groups are more likely than other population groups to live in neighborhoods that might be unsafe or not conducive to healthy living (11,27).

Inconvenient hours was the most frequently reported challenge in previous healthy living programs, which is imperative to consider since timing is a major motivator for male participation in lifestyle change programs (28). The second most frequently reported challenge, lack of motivation, could be mitigated by including more appealing program elements for men and by identifying healthy coping strategies that support lifestyle change. Health coaching that emphasizes accountability and motivation can encourage men’s participation in diabetes management programs (11) and is a key component of lifestyle change programs (2). The program being too costly was the third most frequently reported challenge, which is known to be a hindrance to adopting a healthy lifestyle (6,23,29). Offering diabetes prevention and management programs for free or at reduced cost could potentially eliminate this barrier for many men.

For the men who exercised consistently, having fitness goals and a convenient low-cost routine were key facilitators. This finding points to a need for flexible, relevant, and affordable options that can help men overcome barriers to maintaining healthy habits. More than 40% of the men surveyed in our study cited lack of motivation as a barrier to maintaining a physical activity routine. Since lack of motivation was also mentioned as a challenge in previous healthy living programs, it is important to consider the adverse effect of factors such as psychosocial stress on managing the requirements for healthy lifestyle change (30). Support for such stress could be tailored to address male-specific challenges, like chronic stress related to male gender-role strain (31,32) — which might help men initiate and maintain physical activity. Adequate social support is an important facilitator for the ability to manage one’s health in addition to overcoming extenuating circumstances that might make healthy living challenging.

In the multivariate model, one of the strongest predictors of interest in a program was being physically active; physically active men were twice as likely to be interested in program participation compared with men who were not. Surprisingly, men aged 25 to 44 years were significantly more interested in participating in a healthy living program than those aged 65 years or older. This finding contrasts with the reported lower likelihood of enrollment and retention in the National DPP LCP for people in this age range (24). Innovative strategies to recruit younger and physically active men could increase their enrollment and participation in lifestyle change programs. Another unique finding was that men were more likely to be interested in participating in a healthy living program if they had previously participated in one. These men were motivated to return to a health improvement program, which might suggest the benefit of a trial period, wherein participants can try a program before fully committing. Additionally, such men could be ideal candidates for program champions. Program champions are trusted community members who have successfully made changes and overcome barriers and thereby champion the program for others (33).

### Strengths and limitations

Our study has several strengths. It was a large survey that included a range of responses from men in racial and ethnic minority groups who have historically been underrepresented in and/or excluded from the literature. We captured data on the interests of men on various programmatic elements as well as their health practices, findings that have practical implications for designing and implementing programs to improve men’s health. Through our multivariate model, we identified characteristics of men who are more likely to have interest in participating in a health improvement program. This information will be beneficial for future recruitment, marketing, and retention efforts that focus on improving the health of men in racial/ethnic minority groups.

Our study also has several limitations. The data were self-reported, which could have led to recall or social desirability bias. For example, a higher proportion of the survey respondents reported being physically active (>50%) compared with the national average (31%) (34), so they may have been inclined to respond positively to participating in a healthy living program. Also, the survey population had higher educational attainment than the national average; and since higher educational attainment and health literacy are associated with program participation, the responses of men in this survey may not reflect men with lower educational attainment (35). The interpretation of our cross-tabulation data was limited by the small sample sizes for some of the groups in the survey. Since the study was not designed to test cross-group differences, further interpretation is beyond the scope of this study. Programs and future research could consider other factors (eg, socioeconomic) that might also affect participation, which was beyond the scope of this work. Although we surveyed a large group of men in racial and ethnic minority groups, we acknowledge the limitations of a nonprobability sample and that our sample was not nationally representative or representative of the diversity among these populations.

### Conclusion

Men in racial and ethnic minority groups, who had or were at risk for type 2 diabetes, expressed interest in a program to improve their health and indicated a preference for specific programmatic characteristics in this survey. Programs that add, bolster, or market some of the elements highlighted in our findings could lead to increased numbers of men in racial and ethnic minority groups who participate in programs to improve their health and adopt healthy lifestyle habits. This ultimately could lead to a reduction in the racial and ethnic disparities in the prevalence of diabetes among men.

## References

[1] Centers for Disease Control and Prevention. National Diabetes Statistics Report. May 15, 2024. Accessed November 19, 2024. https://www.cdc.gov/diabetes/php/data-research

[2] Powers MA , Bardsley JK , Cypress M , Funnell MM , Harms D , Hess-Fischl A , . Diabetes Self-management Education and Support in Adults With Type 2 Diabetes: A Consensus Report of the American Diabetes Association, the Association of Diabetes Care and Education Specialists, the Academy of Nutrition and Dietetics, the American Academy of Family Physicians, the American Academy of PAs, the American Association of Nurse Practitioners, and the American Pharmacists Association. *J Acad Nutr Diet.* 2021;121(4):773–788.e9. 10.1016/j.jand.2020.04.020 34924170

[3] Albright AL , Gregg EW . Preventing type 2 diabetes in communities across the U.S.: the National Diabetes Prevention Program. *Am J Prev Med.* 2013;44(4 suppl 4):S346–S351. 10.1016/j.amepre.2012.12.009 23498297 PMC4539613

[4] Mendez I , Lundeen EA , Saunders M , Williams A , Saaddine J , Albright A . Diabetes self-management education and association with diabetes self-care and clinical preventive care practices. *Sci Diabetes Self Manag Care.* 2022;48(1):23–34. 10.1177/26350106211065378 35023406 PMC10979825

[5] Jackson MC , Dai S , Skeete RA , Owens-Gary M , Cannon MJ , Smith BD , . An examination of gender differences in the National Diabetes Prevention Program’s lifestyle change program. *Diabetes Educ.* 2020;46(6):580–586. 10.1177/0145721720964585 33063641 PMC7802597

[6] Hawkins J , Watkins DC , Kieffer E , Spencer M , Espitia N , Anderson M . Psychosocial factors that influence health care use and self-management for African American and Latino men with type 2 diabetes: an exploratory study. *J Men’s Stud.* 2015;23(2):161–176. 10.1177/1060826515582495

[7] Sinclair K , Carty C , Gonzales K , Nikolaus C , Gillespie L , Buchwald D . Strong men, strong communities: design of a randomized controlled trial of a diabetes prevention intervention for American Indian and Alaska Native men. *Am J Men Health.* 2020;14(4):1557988320945457. 10.1177/1557988320945457 32757825 PMC7412907

[8] Friedman DB , Hooker SP , Wilcox S , Burroughs EL , Rheaume CE . African American men’s perspectives on promoting physical activity: “We’re not that difficult to figure out!”. *J Health Commun.* 2012;17(10):1151–1170. 10.1080/10810730.2012.665424 22808914 PMC3504165

[9] Cherrington A , Ayala GX , Scarinci I , Corbie-Smith G . Developing a family-based diabetes program for Latino immigrants: do men and women face the same barriers? *Fam Community Health.* 2011;34(4):280–290. 10.1097/FCH.0b013e31822b5359 21881415 PMC5913741

[10] Griffith DM , King A , Ober Allen J . Male peer influence on African American men’s motivation for physical activity: men’s and women’s perspectives. *Am J Men Health.* 2013;7(2):169–178. 10.1177/1557988312465887 23160732 PMC4145674

[11] Crabtree K , Sherrer N , Rushton T , Willig A , Agne A , Shelton T , . Diabetes Connect: African American men’s preferences for a community-based diabetes management program. *Diabetes Educ.* 2015;41(1):118–126. 10.1177/0145721714557043 25367259 PMC5166559

[12] Hurt TR , Seawell AH , O’Connor MC . Developing effective diabetes programming for Black men. *Glob Qual Nurs Res.* 2015;2:2333393615610576. 10.1177/2333393615610576 28462319 PMC5342290

[13] US Census Bureau Population Division. Annual estimates of the resident population by sex, age, race alone or in combination, and Hispanic origin for the United States: April 1, 2010 to July 1, 2019 (NC-EST2019-ASR5H). 2020. Accessed July 23, 2024. https://www.census.gov/newsroom/press-kits/2020/population-estimates-detailed.html

[14] Centers for Disease Control and Prevention. Behavioral Risk Factor Surveillance System survey data 2019. Accessed July 5, 2022. https://www.cdc.gov/brfss/annual_data/2019/pdf/codebook19_llcp-v2-508.HTML

[15] Ely EK , Gruss SM , Luman ET , Gregg EW , Ali MK , Nhim K , . A national effort to prevent type 2 diabetes: participant-level evaluation of CDC’s National Diabetes Prevention Program. *Diabetes Care.* 2017;40(10):1331–1341. 10.2337/dc16-2099 28500215 PMC5606310

[16] Strawbridge LM , Lloyd JT , Meadow A , Riley GF , Howell BL . Use of Medicare’s diabetes self-management training benefit. *Health Educ Behav.* 2015;42(4):530–538. 10.1177/1090198114566271 25616412

[17] Centers for Disease Control and Prevention. Adjusting Program Delivery During the COVID-19 Public Health Emergency Webinar. August 4, 2022. Accessed November 18, 2024. https://nationaldppcsc.cdc.gov/s/article/Adjusting-Program-Delivery-During-the-COVID-19-Public-Health-Emergency-Webinar

[18] Odom J , Beauchamp C , Fiocchi C , Eicken M , Stancil M , Turner J , . Rapid innovation in diabetes care during Covid-19. *ADCES Pract.* 2020;8(6):28–32. 10.1177/2633559X20951168 38603025 PMC8287092

[19] Hulbert LR , Michael SL , Charter-Harris J , Atkins C , Skeete RA , Cannon MJ . Effectiveness of incentives for improving diabetes-related health indicators in chronic disease lifestyle modification programs: a systematic review and meta-analysis. *Prev Chronic Dis.* 2022;19:E66. 10.5888/pcd19.220151 36302383 PMC9616129

[20] Realmuto L , Kamler A , Weiss L , Gary-Webb TL , Hodge ME , Pagán JA , . Power Up for Health-Participants’ Perspectives on an Adaptation of the National Diabetes Prevention Program to Engage Men. *Am J Men Health.* 2018;12(4):981–988. 10.1177/1557988318758786 29540130 PMC6131458

[21] Walker EA , Weiss L , Gary-Webb TL , Realmuto L , Kamler A , Ravenell J , . Power Up for Health: pilot study outcomes of a diabetes prevention program for men from disadvantaged neighborhoods. *Am J Men Health.* 2018;12(4):989–997. 10.1177/1557988318758787 29540129 PMC6131473

[22] Frediani JK , Bienvenida AF , Li J , Higgins MK , Lobelo F . Physical fitness and activity changes after a 24-week soccer-based adaptation of the U.S diabetes prevention program intervention in Hispanic men. *Prog Cardiovasc Dis.* 2020;63(6):775–785. 10.1016/j.pcad.2020.06.012 32603753 PMC8650220

[23] Cavanaugh CL , Taylor CA , Keim KS , Clutter JE , Geraghty ME . Cultural perceptions of health and diabetes among Native American men. *J Health Care Poor Underserved.* 2008;19(4):1029–1043. 10.1353/hpu.0.0083 19029735

[24] Cannon MJ , Masalovich S , Ng BP , Soler RE , Jabrah R , Ely EK , . Retention Among Participants in the National Diabetes Prevention Program Lifestyle Change Program, 2012-2017. *Diabetes Care.* 2020;43(9):2042–2049. 10.2337/dc19-2366 32616617 PMC11000538

[25] Pillay J , Armstrong MJ , Butalia S , Donovan LE , Sigal RJ , Vandermeer B , . Behavioral programs for type 2 diabetes mellitus: a systematic review and network meta-analysis. *Ann Intern Med.* 2015;163(11):848–860. 10.7326/M15-1400 26414227

[26] Andrade ACS , Mingoti SA , Fernandes AP , Andrade RG , Friche AAL , Xavier CC , . Neighborhood-based physical activity differences: Evaluation of the effect of health promotion program. *PLoS One.* 2018;13(2):e0192115. 10.1371/journal.pone.0192115 29401506 PMC5798787

[27] US Department of Health and Human Services. Healthy People 2030. Neighborhood and built environment. Accessed August 6, 2024. https://odphp.health.gov/healthypeople/objectives-and-data/browse-objectives/neighborhood-and-built-environment

[28] Gary-Webb TL , Walker EA , Realmuto L , Kamler A , Lukin J , Tyson W , . Translation of the National Diabetes Prevention Program to engage men in disadvantaged neighborhoods in New York City: a description of Power Up for Health. *Am J Men Health.* 2018;12(4):998–1006. 10.1177/1557988318758788 29540131 PMC6131470

[29] Rutledge S , Hulbert L , Charter-Harris J , Smith A , Owens-Gary M . A qualitative exploration of facilitators and barriers to adopting a healthy lifestyle among Black, Hispanic, and American Indian males with diabetes or at risk for type 2 diabetes. *Ethn Health.* 2024;29(4–5):447–464. 10.1080/13557858.2024.2359377 38842432

[30] Owens-Gary MD , Zhang X , Jawanda S , Bullard KM , Allweiss P , Smith BD . The importance of addressing depression and diabetes distress in adults with type 2 diabetes. *J Gen Intern Med.* 2019;34(2):320–324. 10.1007/s11606-018-4705-2 30350030 PMC6374277

[31] Griffith DM , Gunter K , Allen JO . Male gender role strain as a barrier to African American men’s physical activity. *Health Educ Behav.* 2011;38(5):482–491. 10.1177/1090198110383660 21632436 PMC4381925

[32] Hawkins J , Watkins DC , Kieffer E , Spencer M , Piatt G , Nicklett EJ , . An exploratory study of the impact of gender on health behavior among African American and Latino men with type 2 diabetes. *Am J Men Health.* 2017;11(2):344–356. 10.1177/1557988316681125 27923970 PMC5675282

[33] Centers for Disease Control and Prevention. Program champion strategy toolkit. 2022. Accessed February 24, 2023. https://nccdphp.my.salesforce.com/sfc/p/#t0000000TZNF/a/3d000000Aotu/ClqU.JBkCt7qGHUIkpxNT.h4.rPjGwb2Kyia0w1bTmg

[34] Hyde ET , Whitfield GP , Omura JD , Fulton JE , Carlson SA . Trends in meeting the physical activity guidelines: muscle-strengthening alone and combined with aerobic activity, United States, 1998-2018. *J Phys Act Health.* 2021;18(S1):S37–S44. 10.1123/jpah.2021-0077 34465652 PMC11000248

[35] National Center for Education Statistics. Degrees conferred by race/ethnicity and sex. 2020. Accessed February 28, 2023. https://nces.ed.gov/fastfacts/display.asp?id=72

